# Establishment of primary human breast cancer cell lines using “pulsed hypoxia” method and development of metastatic tumor model in immunodeficient mice

**DOI:** 10.1186/s12935-019-0766-5

**Published:** 2019-02-28

**Authors:** Anna A. Nushtaeva, Anastasia A. Karpushina, Mikhail S. Ermakov, Ludmila F. Gulyaeva, Alexey V. Gerasimov, Sergey V. Sidorov, Tatyana A. Gayner, Anastasia Y. Yunusova, Anastasia V. Tkachenko, Vladimir A. Richter, Olga A. Koval

**Affiliations:** 1 Institute of Chemical Biology and Fundamental Medicine, Siberian Branch, Russian Academy of Sciences, Lavrentiev Avenue, 8, Novosibirsk, 630090 Russia; 2 Novosibirsk State University, Pirogova Str. 1, Novosibirsk, 630090 Russia; 3 Institute of Molecular Biology and Biophysics, Siberian Branch, Russian Academy of Medical Sciences, Ac. Timakov Str. 2, Novosibirsk, 630117 Russia; 4 National Novosibirsk Regional Oncology Dispensary, Plakhotnogo Str. 2, Novosibirsk, 630000 Russia; 5 Novosibirsk Municipal Budgetary Healthcare Institution “Municipal Clinical Hospital #1”, Zalessky Str. 6, Novosibirsk, 630047 Russia

**Keywords:** Breast cancer, Primary cultures, EMT, MET, Pulsed hypoxia, E-cadherine, Mice tumor models, Mediastinum lymph node metastasis

## Abstract

**Background:**

Among breast cancer (BC) patients the outcomes of anticancer therapy vary dramatically due to the highly heterogeneous molecular characteristics of BC. Therefore, an extended panel of BC cell lines are required for in vitro and in vivo studies to find out new characteristic of carcinogenesis and metastasis. The purpose of this study was to develop patient-derived BC cell cultures and metastatic tumor models representing a tool for personal therapy and translational research.

**Methods:**

Breast cancer cells were prepared by optimizing technique from tumor samples. We used real-time RT-PCR, flow cytometry, western blotting, cytotoxicity assay, karyotyping and fluorescent and electron microscopy analyses to characterize the established cell lines. BC xenografts in *scid* mice were used for in vivo tumorigenicity studies.

**Results:**

The technique of preparing primary cells was optimized and this resulted in a high output of viable and active proliferated cells of nine patient-derived breast cancer cell lines and one breast non-malignant cell line. High E-cadherine and EpCAM expression correlated positively with epithelial phenotype while high expression of N-cadherine and Vimentin were shown in cells with mesenchymal phenotype. All mesenchymal-like cell lines were high HER3-positive—up to 90%. More interesting than that, is that two cell lines under specific culturing conditions (pulsed hypoxia and conditioned media) progressively transformed from mesenchymal to epithelial phenotypes displaying the expression of respective molecular markers proving that the mesenchymal-to-epithelial transition occurred. Becoming epithelial, these cells have lost HER3 and decreased HER2 membrane receptors. Three of the established epithelial cancer cell lines were tumorigenic in SCID mice and the generated tumors exhibited lobules-like structures. Ultrastructure analysis revealed low-differentiate phenotype of tumorigenic cell lines. These cells were in near-triploid range with multiple chromosome rearrangements. Tumorigenic BrCCh4e cells, originated from the patient of four-course chemotherapy, initiated metastasis when they were grafted subcutaneous with colonization of mediastinum lymph nodes.

**Conclusions:**

The developed BC cells metastasizing to mediastinum lymph nodes are a relevant model for downstream applications. Moreover, our findings demonstrate that pulsed hypoxia induces transformation of primary fibroblastoid breast cancer cells to epithelial-like cells and both of these cultures—induced and original—don’t show tumor initiating capacity.

**Electronic supplementary material:**

The online version of this article (10.1186/s12935-019-0766-5) contains supplementary material, which is available to authorized users.

## Background

Breast cancer is a highly heterogenic oncological disease initially classified into five intrinsic subtypes based solely on gene expression of estrogen receptors (ERs), progesterone receptor (PGR) and tyrosine kinase receptor HER2 [1]. A wide spectrum of therapeutics including targeted drugs are applied for the treatment of BC based on a indicated subtype. However, the mortality rate for advanced BC is stable and high, and the majority of breast cancer related deaths are the result of a metastatic spread of the cancer cells. Although a vast number of immortalized human breast cancer cell lines being injected into immunocompromised animals induce the growth of tumor nodes, only a few of them induce metastases [2, 3]. Such limitation of cultured cancer cells becomes complicated in the investigation of metastasis and metastasis-associated cellular transformation in vivo as well as the anti-metastatic potential of new therapeutics. Therefore, the development of the new models of BC and its metastasis in vivo are still significant. Bioptats and tumor tissues specimens are the source for developing new BC cell lines which are primary patient-derived cultures at the initial stage of passaging. Patient-specific cell lines allow us to realize personal therapy as well as to find out new characteristic of carcinogenesis and metastasis. This approach can give the direct information of the tumor cell’s sensitivity to defined drugs in contrast to the prediction methods based on genome-wide genotyping data [4].

Morphological changes in BC cells can be induced in vitro by various molecular (TGF-β1, EGF, IL-6) or cellular (co-cultivation with adipocytes or stromal fibroblasts) stimulus producing cells with epithelial or fibroblastoid phenotype [5–8]. Association of epithelial–mesenchymal transition (EMT) and reverse mesenchymal-epithelial transition in breast cancers with tumor-initiating capacity, metastasis and chemoresistance make these transitions highly important in BC biology. Epithelial or mesenchymal phenotype of breast cancer cells usually correlates with the expression of special markers, for example N-cadherin (N-cad), E-cadherine (E-cad) and Vimentin [9]. E-cadherin is essential for the integrity of epithelial tissue while N-cad downregulates E-cad to undergo EMT finally leading to the enhancement of motility of mammary epithelial cell lines [10].

We herein describe the modified method for primary breast cancer cell cultures development, their molecular characterization and MET induction either tumorigenesis analysis. Additionally, we established a cell model for producing mediastinum lymph nodes metastasis in mice.

## Materials and methods

### Chemicals and antibodies

Cisplatin, doxorubicin, everolimus (afinitor) were purchased from Sigma-Aldrich (St. Louis, MO, USA). Phycoerythrin (PE)-conjugated mouse anti-human CD44 monoclonal (#MHCD4404) and fluorescein isothiocyanate (FITC)-conjugated mouse anti-human CD24 monoclonal (#MHCD4201) antibodies were purchased from Molecular Probes (Invitrogen, Carlsbad, CA, USA). FITC-conjugated mouse anti-human HER2 monoclonal (#2222020), FITC-conjugated mouse anti-human CD326 monoclonal (#2221020), PE -conjugated mouse anti-human CD324 monoclonal (#2220530), PE -conjugated mouse anti-human CD325 monoclonal (#2354025), allophycocyanin (APC)-conjugated mouse anti human HER3 monoclonal (#2223540), APC-conjugated mouse anti human CD146 monoclonal (#2310060) antibodies were purchased from Sony Biotechnology Inc. (San Jose, CA, USA). FITC-, APC-, and PE-conjugated IgG controls were from BD Biosciences. Vimentin mouse monoclonal (ab8069) and rabbit polyclonal to Ki-67 (ab15580) antibodies were from Abcam (Cambridge, United Kingdom). Secondary antibodies Donkey Anti-Mouse IgG H&L (ab97029, Abcam), Alexa Fluor^®^ 594 goat anti-rabbit IgG1 (Thermo Fisher Scientific), goat anti-mouse HRP-conjugated polyclonal IgG (Abcam) and donkey anti-goat HRP-conjugated IgG (R&Dsystems) were used.

### Cell cultures

MCF7, MDA-MB-231, and SKOV3 cells were obtained from the Russian cell culture collection (Russian Branch of the ETCS, St. Petersburg, Russia). MDA-MB-231 cells were grown in Leibovitz media (L15, Sigma-Aldrich) supplemented with 10% fetal bovine serum (FBS; Gibco BRL Co., Gaithersburg, MD, USA), 2 mM l-glutamine, 250 mg/mL amphotericin B, and 100 U/mL penicillin/streptomycin. MCF7 cells were cultivated in Iscove’s modified Dulbecco’s media (IMDM; Sigma-Aldrich) with 10% FBS (Gibco BRL Co., Gaithersburg, MD, USA), 2 mM l-glutamine (Sigma-Aldrich), 250 mg/mL amphotericin B, and 100 U/mL penicillin/streptomycin (Gibco BRL Co., Gaithersburg, MD, USA). SKOV3 cells were grown in Dulbecco’s Modified Eagle Medium: Nutrient Mixture F-12 (DMEM:F12, Sigma-Aldrich) supplemented with 10% fetal bovine serum (FBS; Gibco BRL Co., Gaithersburg, MD, USA), 2 mM l-glutamine, 250 mg/mL amphotericin B, and 100 U/mL penicillin/streptomycin.

### Human tissue specimens

All tissue samples (normal and tumor) were obtained with written informed consent from patients at the Novosibirsk Municipal Budgetary Healthcare Institution “Municipal Clinical Hospital №1” (Novosibirsk, Russian Federation) and National Novosibirsk Regional Oncology Dispensary. The final diagnosis of cancer was confirmed by hematoxylin and eosin staining of paraffin blocks after the surgery (Table 1). One tissue sample was obtained from healthy woman during size-reduction plastic surgery (Centre of New Medical Technologies, Novosibirsk, Russian Federation). The study protocol was approved by the Institute of Molecular Biology and Biophysics SB RAS Ethics Committee (Report#1 from March, 14 2017) in accordance with the World Medical Assosiation Declaration of Helsinki. Fresh tumor and normal tissue specimens were immediately transferred into ice-cold DMEM medium (Gibco BRL Co., Invitrogen) supplemented with 100 U/mL penicillin, 100 μg/mL streptomycin, and 250 mg/mL amphotericin B.

**Table 1:** Features of tissue specimens and established cells

Primary culture (method-dependent)	Morphology		Tissue specimens
	Collagenase dissociation	Collagenase-free	
BrC3	f	e	Bl mammae T1N0M0
BrC4	f	–	Bl mammae T2N0M0
BrC5	f	–	Bl mammae T3N0M0
BrC6	f	–	Bl mammae T2N1M0
BrCCh2	f	e	Bl mammae T2N0M0 4 courses of chemotherapy
BrCCh3	f	f	Bl mammae T1N0M0 4 courses of chemotherapy
BrCCh4	–	e	Bl mammae T4N1M0 4 courses of chemotherapy
BN4	f	–	Normal cells

4 courses of chemotherapy included doxorubicin/cyclophosphamide before surgery

*f* fibroblastoid-like morphology, *e* epithelial-like morphology

### Primary cell culture preparation

Breast tumor tissue was isolated and processed in a sterile manner. Tissues were washed extensively with 1× PBS with 200 U/mL penicillin, 200 μg/mL streptomycin, and 500 mg/mL amphotericin without centrifugation.

For collagenase treatment tissue specimens were mechanically dissociated using a scalpel with removal of vascular material and transferred to a solution of 20 mg/mL collagenase I (Gibco BRL Co., Invitrogen) in DMEM media and incubated at 37 °C for 15 h on a shaking incubator (Grant Bio, Keison Products, UK). Specimens dissociated into single cells were washed with 10× excess of phosphate-buffered saline (PBS) and cell pellet was collected by centrifugation at 300×*g* for 5 min. Cells were plated in IMDM with 10% FBS and, after cell adhesion, 10 μM Rho-associated protein kinase (ROCK) inhibitor was added to the culture medium for 1 h. Next, the media was replaced with fresh complete IMDM media. At the next passages, cells were cultured in complete IMDM media supplemented with epithelial cell growth supplement (#6622, Cell Biologics, Chicago, IL, USA), Mito + Serum Extender (BD Biosciences—Discovery Labware, San Jose, CA, USA), 2 mM l-glutamine, 100 U/mL penicillin, 100 μg/mL streptomycin, and 250 mg/mL amphotericin B and were cultivated in 6-well plates at 37 °C in a humidified atmosphere containing 5% CO_2_. When 70–80% confluence was reached, cells were harvested using 0.05% trypsin/ethylenediaminetetraacetic acid (Sigma-Aldrich) and sub-cultured for further experiments.

In the case of collagenase-free method, mechanically dissociated tissue specimens were put into IMDM media with 10% FBS, supplemented with Mito + Serum Extender (BD Biosciences–Discovery Labware, San Jose, CA, USA), 2 mM l-glutamine, 100 U/mL penicillin, 100 μg/mL streptomycin, and 250 mg/mL amphotericin B and were cultivated in 6-well plates at 37 °C in a humidified atmosphere containing 5% CO_2_. Every 36 h culture media with detached cells was transfer to new well, and portions fresh media were added to new well and to initial well also. This manipulation was repeated 2–3 times to stimulate cell division. Cells were detached by TripLE™ (Gibco BRL Co., Invitrogen) when reached a monolayer.

### MTT assay

The cytotoxic effects of the cisplatin, doxorubicin, and everolimus (afinitor) on human tumor cells were investigated using the MTT assay (Sigma-Aldrich; Merck Millipore) according to a protocol described previously. The cells that had reached 30% confluence in a 96-well plate were incubated for 48 h with preparations at various concentrations. After incubation, the supernatant was removed and 200 µL MTT solution in RPMI 1640 medium (0.5 mg/mL) was added to each well and incubated for 4 h at 37 °C. The formazan crystals were dissolved in 150 µL dimethyl sulfoxide. The optical density of the formazan solutions was measured using an Apollo LB912 photometer (Berthold Technologies, Oak Ridge, TN, USA) at a wavelength of 570 nm. Cell viability was determined relative to the viability of the control cells (100%) ± standard deviation in three independent experiments.

### Flow cytometry

Cells growing in 6-well plates were collected, fixed in 10% neutral buffered formalin (BioVitrum, Russia), and incubated with labeled mouse anti-human antibodies (CD24, CD44 and CD133 or CD325, HER2/CD340 and HER3, CD146, CD 324 and CD326) in PBS supplemented with 10% normal goat serum for 30 min in ice. All analyses were performed using a FACSCantoII flow cytometer (BD Biosciences, Franklin Lakes, NJ, USA), and the data were analysed by FACSDiva Software (BD Biosciences). Cells were initially gated based on forward versus side scatter to exclude small debris, and ten thousand events from this population were collected. Control cells were treated with appropriate isotype PE-, FITC- and APC-conjugated IgG (BD Biosciences).

### Immunocytochemistry

Cells (1 × 10^4^) growing in four-well culture slides (BD Falcon, Bedford, MA) were fixed with ice cold methanol. For permeabilization, 0.1% Triton X-100 was added to cells for 10 min. To block nonspecific antibody binding, cells were incubated in 1% BSA (Sigma-Aldrich), 0.3 M glycine in PBST buffer (PBS with 0.1% Tween 20) for 30 min at RT. Next, cells were incubated with anti-vimentin and anti-Ki-67 antibodies for 40 min at RT. For visualization FITC-conjugated and Alexa Fluor^®^ 594-conjugated secondary antibodies were used for 1 h at RT. Stained cells were visualized using an Axioscop 2 PLUS fluorescence microscope (Carl Zeiss, GmbH).

### Western blot analysis

Tumor cells were lysed in buffer: 50 mM Tris (pH 8.0), 5 mM EDTA, 150 mM NaCl containing 0.1% SDS, 1× complete protease inhibitor cocktail (Roche Diagnostics GmbH, Mannheim, Germany) and 1 mM PMSF. Samples (30 μg) were separated by 10% SDS-PAGE and transferred to a Trans-Blot nitrocellulose membrane (Bio-Rad Laboratories) by a wet blotting procedure (100 V, 500 mA, 90 min, 15 °C) using “Mighty Small Transphor” (GE healthcare Bio-Science AB, USA). Immunodetection was performed using iBind system (Life Technologies), iBind Cards (Invitrogen, Thermo Fisher Scientific) and antibodies: Vimentin mouse monoclonal antibody (1:50), GAPDH goat polyclonal antibodies (1:133), goat anti-mouse HRP-conjugated polyclonal IgG (1:200, Abcam) or donkey anti-goat HRP-conjugated IgG (1:200, R&D systems) as secondary antibodies, with Novex ECL HRP chemiluminescent substrate reagent kit (Invitrogen, USA). A C-DiGit blot scanner (Li-COR Bioscience) was used for luminescent detection. Densitometric analysis of the western blot data was performed using the image analysis software Gel-Pro Analyser (Media Cybernetics) version 3.1.

### Electron microscopy

Cells that had reached 80–90% confluence were washed with PBS and detached with 10 mM EDTA/PBS (pH 8.0). Then, cells were washed with PBS and fixed with 4% paraformaldehyde solution. Fixed precipitates were washed with the IMDM media, post-fixed with 1% solution of osmium tetroxide and dehydrated. Next, Epon-Araldite (SPI, USA) was added to the mixture. Ultrathin sections were obtained from the resulting blocks on an EM UC7 ultramicrotome (Leica, Germany). Sections were contrasted with uranyl acetate solution and lead citrate and examined in a JEM 1400 transmission electron microscope (JEOL, Japan) equipped with a Veleta side-input digital camera (Olympus Soft Imaging Solution, Germany).

### Real-time PCR

Total cellular RNA was isolated using the Lira reagent (Biolabmix Ltd., Novosibirsk, Russia) according to the manufacturer’s protocol. RT-PCR reaction was performed with BioMaster RT-PCR SYBR Blue (Biolabmix, Novosibirsk, Russia) by LightCycler 96 System (Roche, Basel, Switzerland). Gene-specific primers were used: ERα: 5′-ATGATGAAAGGTGGGATACGA-3′ and 5′-CTGTTCTTCTTAGAGCGTTTGATC-3′; PGR: 5′-TCATTCTATTCATTATGCCTTACCA-3′ and 5′-GACTTCGTAGCCCTTCCAAAG-3′; Cyp19: 5′-TGCGAGTCTGGATCTCTGGA-3′ and 5′-GGGCCTGACAGAGCTTTCATA-3′; hypoxanthineguanine phosphoribosyltransferase (HPRT): 5′-CATCAAAGCACTGAATAGAAAT-3′ and 5′-TATCTTCCACAATCAAGACATT-3′; glyceraldehyde 6-phosphate dehydrogenase (GAPDH): 5′-GAAGATGGTGATGGGATTTC-3′ and 5′-GAAGGTGAAGGTCGGAGT-3′. The levels of mRNAs were represented as relative values normalized to the level of GAPDH and HPRT mRNA. The quality of reference genes was assessed using geNorm (Qbase +) [11, 12]. Mean values (± standard deviation) from three independent experiments are shown.

### Karyotyping

Cells at passages 4–5 were growing under standard conditions for 48 h. In order to accumulate mitotic cells in metaphase, 0.1% colchicine solution was added to the cells for 3 h at 37 °C. Hypotonic solution (75 mM KCl) was added to the cells before standard fixation with ethanol:acetic acid (3:1). Next, cells were harvested by trypsin, cellular suspension was dropped on ice-cold microscope slide, dried, and stained with Giemsa (GTG-banding). Slides were analyzed with an Axioscop 2 PLUS (Carl Zeiss, GmbH). Only roundish and individual metaphases were analyzed. About 50–60 metaphase cells from each slide were observed.

### Histomorphological sataining

Tumor specimens (≤ 1 cm^2^) were plated in 10% neutral-buffered formalin. The samples were then processed, paraffin-embedded and sectioned to 4 µm. After deparaffinization and hydrating, the sections were stained with hematoxylin and eosin. The slides were visualized with an Axioscop 2 PLUS (Carl Zeiss, GmbH).

### Animals

Female SCID hairless outbred (SHO-PrkdcscidHrhr) mice aged 6–8 weeks were obtained from the SPF vivarium of the Institute of Cytology and Genetics of the Siberian Branch of the Russian Academy of Science (SB RAS) (Novosibirsk, Russia). Mice were housed in individually ventilated cages (Animal Care Systems, Colorado, USA) in groups of 1–2 animals per cage with ad libitum food (ssniff, Soest, Germany) and water. Mice were kept in the same room within a specific pathogen-free animal facility with a regular 14/10 h light/dark cycle (lights on at 02:00 h) at a constant room temperature of 22 ± 2 °C and relative humidity of approximately 45 ± 15%.

All animal experiments were carried out in compliance with the protocols and recommendations for the proper use and care of laboratory animals (EEC Directive 86/609/EEC). The study protocol was approved by the Committee on the Ethics of Animal Experiments of the Administration of SB RAS (Permit #40 from April, 4 2018).

A suspension of primary cells (3.5–4 × 10^7^ cells/mL) in PBS was mixed with Matrigel (BD Bioscience) at a ratio of 1:1, and 0.1 mL of suspension was injected subcutaneously into the back of each mouse.

### Statistics

Data are expressed as the mean ± standard deviation. Statistical analysis was performed using OriginPro 2015 software. For normal data distribution the significance was determined using a two-tailed Student’s *t* test. For non-normal data distribution, the Mann-Witney u-test was used.

## Results

### Patient-derived cell cultures establishment

**Fig. 1:**
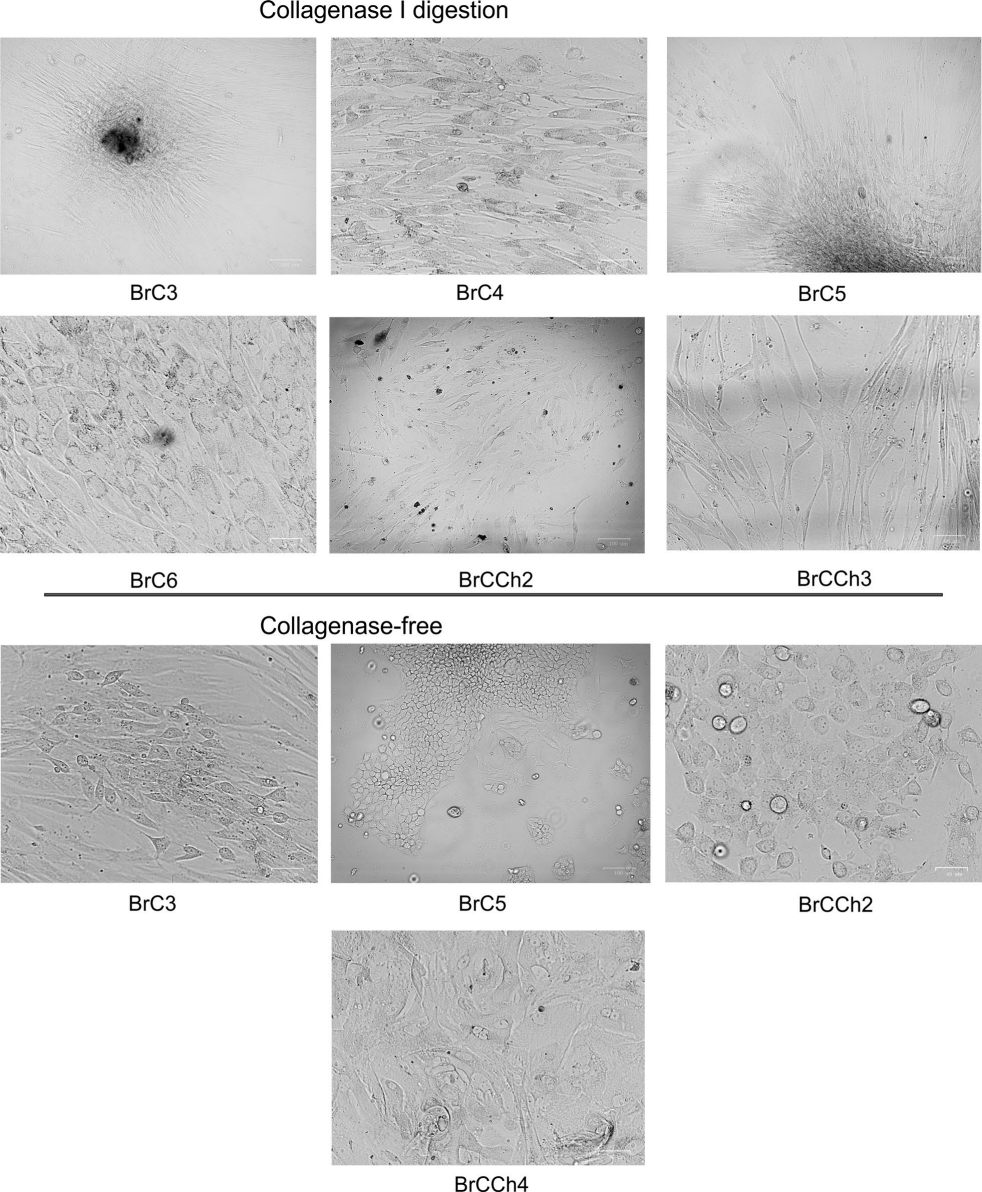
Morphology of primary breast cancer cells in vitro. Cells were prepared by the collagenase I dissociation or by the collagenase-free method, as indicated. Phase-contrast images of cells growing as monolayer at 1–2 passages. Images were captured with ZOE Fluorescent Cell Imager (BioRad)

Seven breast cancer tissues and one normal breast tissue were used for the establishment of primary cultures. Three of seven cancer tissue specimens were from the patients who have received four courses of chemotherapy (with doxorubicin/cyclophosphamide before surgery). The protocol of preparation for patient-derived cells was optimized to obtain high yield of viable epithelial cells. For this purpose, we compared routine treatment with collagenase-I as we previously described [13] and the collagenase-free method for all tissue samples. We observed that the adhesion period of cells prepared by collagenase-free method was 36 h on an average whereas after collagenase dissociation, it was about 72 h. BrC3, BrC5, BrCCh2 and BrCCh4 tissues produced good adhesion cultures by both methods but only the collagenase-free method produced cells with epithelial phenotype (Fig. 1) For BrC4, BrC6, and BrCCh3 tissues cell adhesion was observed only with collagenase method (Fig. 1). All cells obtained by collagenase method predominately demonstrated spindle-like morphology. Features of tissue specimens and established cells are summarized in Table 1. BN normal cells prepared from breast tissue of a healthy donor shows fibroblastoid phenotype.

### Pulsed hypoxia conditions induce transformation of fibroblastoid primary breast cancer cells to epithelial phenotype

**Fig. 2:**
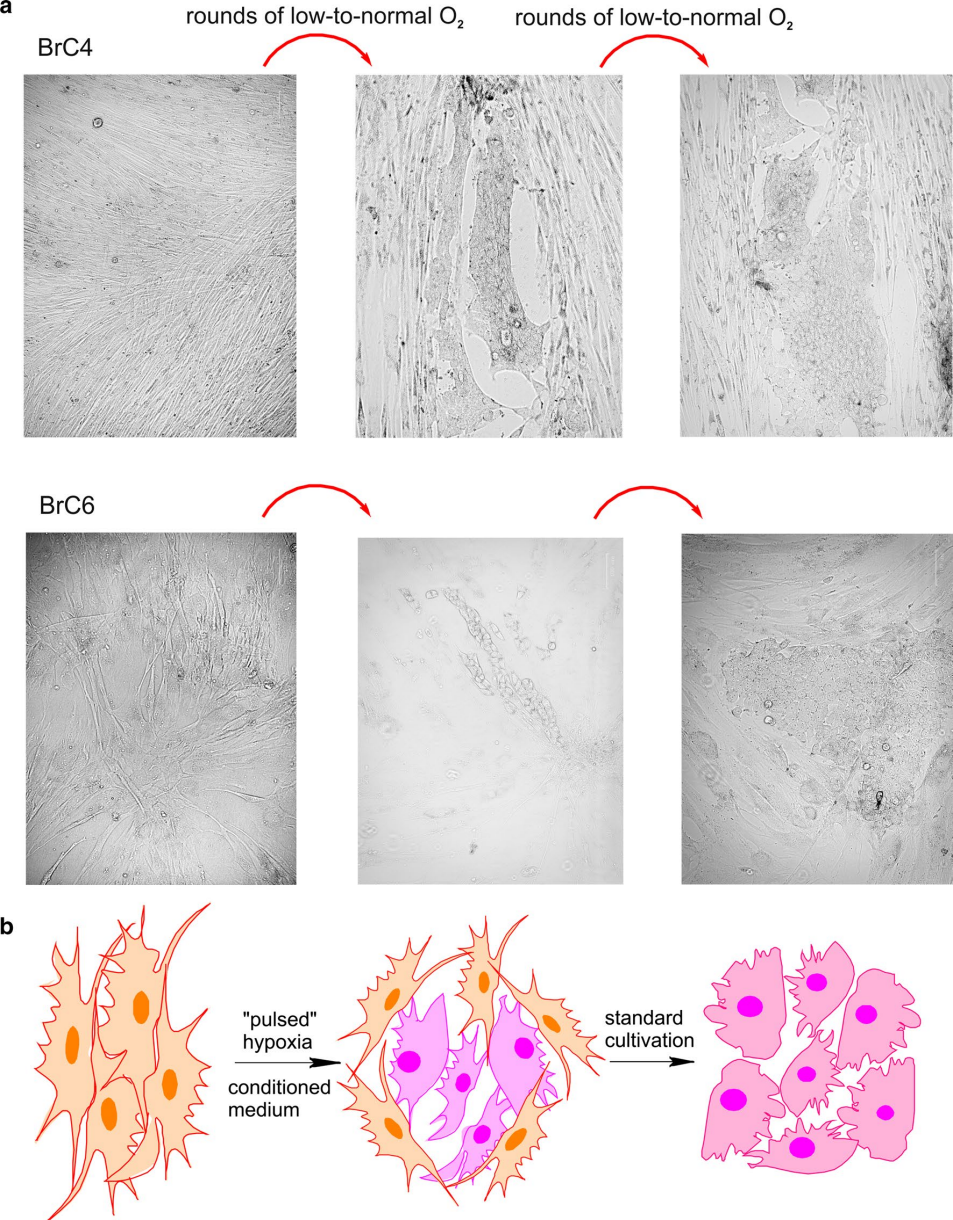
Effects of “pulsed hypoxia” in vitro. **a** Morphology of breast cancer cell transformation, observed during cultivation (phase-contrast microscopy). **b** Scheme of cell culture transformation induced by “pulsed hypoxia”

Having a task to develop a protocol for establishment of epithelial primary cancer cells, we modified cultivation conditions for fibroblastoid-like cells in order to induce the shift of phenotype to epithelioid. Initially, these cells were stayed under normoxia to reach about 80% confluence and then islets of cells with epithelial morphology were estimated. If islets of cells with epithelial morphology consisted of not less than 5–6 cells, cells were subjected to hypoxia (2% O_2_). Four rounds of replacement of the normal to hypoxic conditions and the adding of conditioned medium for BrC4f, BrC6f, and BrCCh3f cells resulted in the epithelial phenotype for BrC4 and BrC6 cells but not for BrCCh3 cells (Fig. 2a). We named such rounds of changes hypoxia to normoxia as “pulsed hypoxia”. Cultivation of BrC4f, BrC6f and BrCCh3f cells with conditioned medium under standard O_2_ concentration did not induce transformation of fibroblastoid cells to epithelial cells. One dose of hypoxia didn’t produce homogeneous epithelial phenotype: if cells stay under normal O_2_ after one round of hypoxia, fibroblastoid cells totally supplant epithelial cells. We observed that not less than 3 rounds of hypoxia lead to epithelial cell culture. Figure 2b demonstrates the scheme of cell culture transformation which we observed during cultivation. Next, under standard cultivation conditions, the phenotypes of parental and transformed cells were stable for no less than 20 passages. We conclude that pulsed hypoxia is crucial for the process shown.

**Fig. 3:**
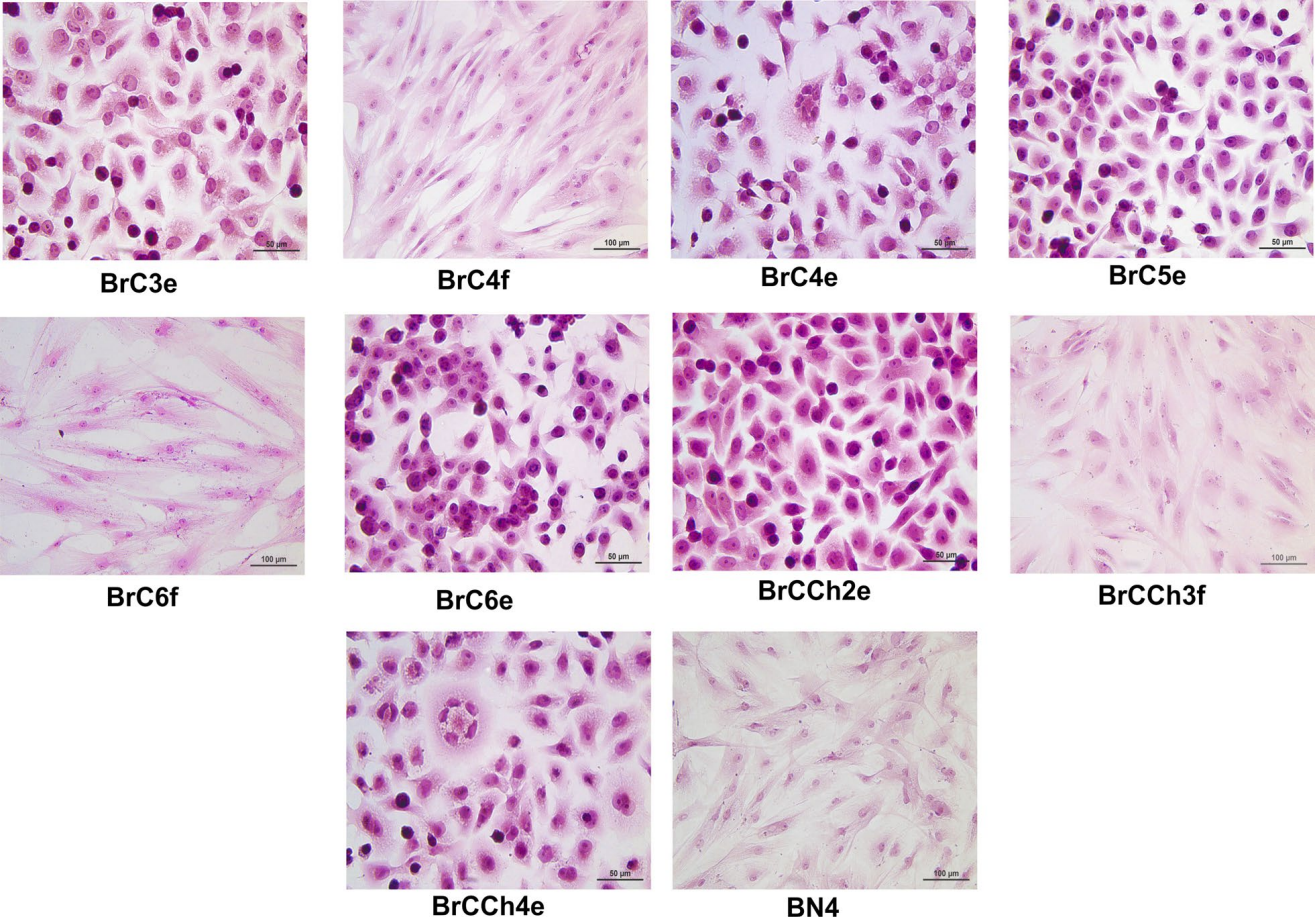
Morphology of growing primary breast cancer and normal cells. Cells were grown on culture slides and then stained with hematoxylin and eosin (4–5 passages)

Thus, BrC4 and BrC6 tumors were presented by pairs of cell cultures: epithelial-like with symbol “e” and fibroblastoid-like cells with symbol “f”. Morphological characteristics of cells that were used for further experiments are shown at Fig. 3.

### Molecular characteristics of established patient-derived cells

To assess whether the established cell cultures express ERα, PGR, and aromatase (Cyp19), their specific mRNAs were compared with mRNA levels in the ER-positive human breast adenocarcinoma cell line MCF-7. Analysis of cells for PGR, ERα and Cyp19 expression was performed by Real Time PCR with specific primers. No differences were detected in mRNAs expression between the investigated cells, and most of the cells were hormone receptors-negative (Additional file 1). Interestingly, that only fibroblastoid-like BrCCh3f and BN4 cells were ERα^+^/PGR^+^ double-positive.

**Fig. 4:**
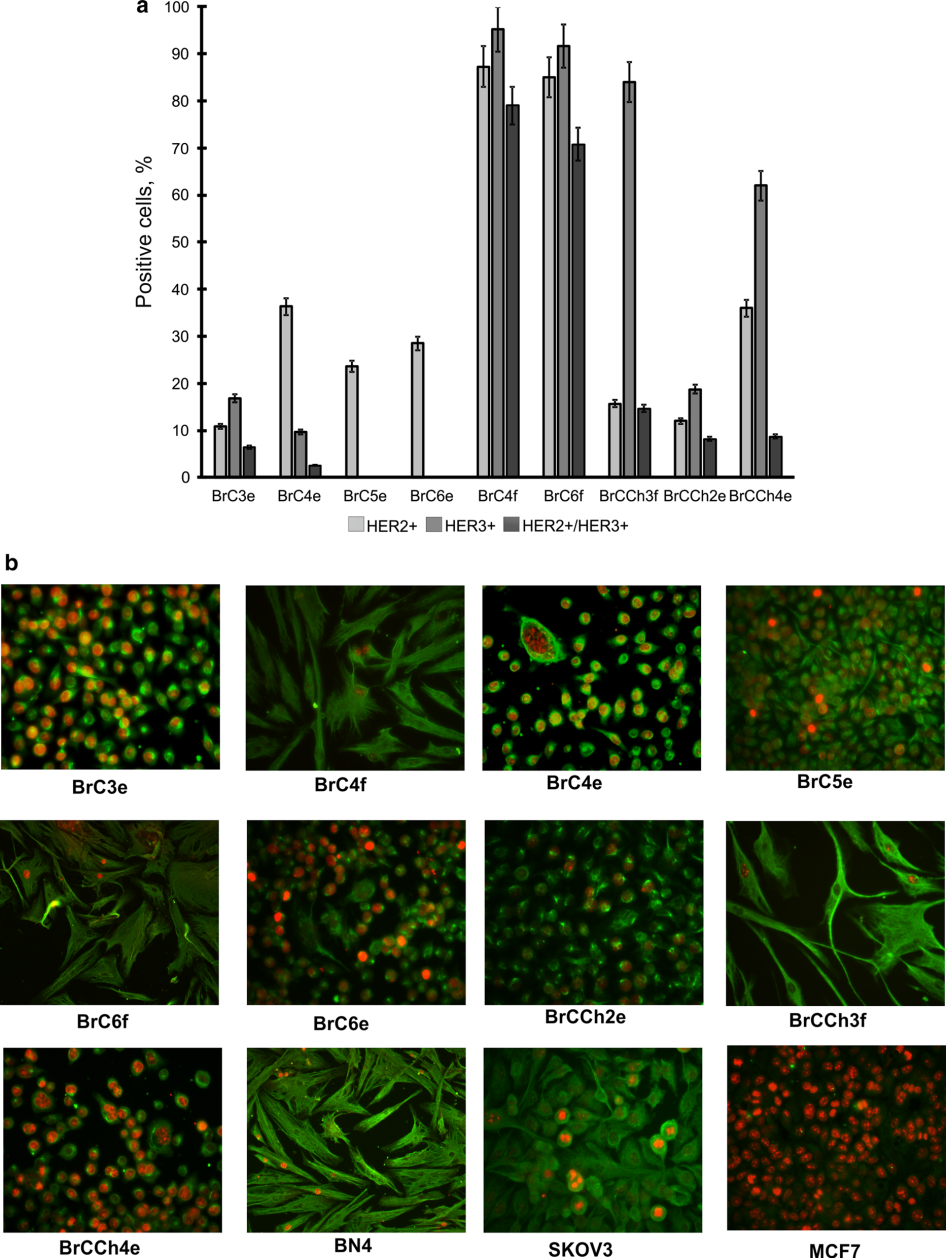
Expression of HER2, HER3, Vimentin and Ki-67 in primary and cultured cells. **a** Analysis of surface expression of HER2 and HER3 by flow cytometry with specific antibodies. **b** Double immunofluorescence staining of Vimentin (green) and Ki-67 (red). Immunocytochemical detection (×40) in cancer and normal human cells

HER2 and HER3 surface expression in established cell lines were analysed by flow cytometry and double-positive HER2^+^/HER3^+^ population were also estimated (Fig. 4a). Positivity for HER2 was defined as more than 10% cells stained for HER2, according to ASCO guideline, and for HER3 the cutoff value was the same [14]. Two groups are easily marked out among investigated cell lines, one of which showed high HER2 and HER3 expression while the other consisted of HER2 +/HER3^−/low^. Group with HER2^high^/HER3^high^ double-positive expression was presented by cells with fibroblastoid morphology—BrC4f and BrC6f. BrC4e, BrC5e and BrC6e cells were HER3-negative with moderate HER2. In turn BrC3e and BrCCh2e cells expressed near-negative HER3 and HER2. Only BrCCh4e cells were HER3-positive among epithelial cells.

### Correlation of mesenchymal markers between established cells with cell phenotype

Since several cell line demonstrated fibroblast-like morphology, the epithelial and mesenchymal markers were detected by flow cytometry and fluorescent microscopy in all the cells. We analyzed expression of Vimentin, E-cadherin, N-cadherin, EpCAM and Mel-cam as well as Ki-67. Human ovary adenocarcinoma SCOV3 cells were used as positive control for Ki-67 and Vimentin and MCF-7 were used as Vimentin^−^/Ki-67^high^ cells. Immunocytochemical analysis demonstrated highest Ki-67 (> 95%) expression in BrC3e and BrCCh4e cells, and all cells with epithelial phenotype were Ki-67-positive, basing on the 2011 St Gallen International Expert Consensus classification (Fig. 4b) [15]. BrC4f and BrC6f cells were Ki-67^low^ (< 10%) that was similar to normal BN4 cells. The mesenchymal marker Vimentin was visualized in all primary cells, but only in fibroblast-like cells did it demarcate the cytoskeletal structure. Staining intensity and distribution of Vimentin varied across the cells. Western-blot analysis of cell lysates shown that only fibroblastoid-like cells expressed well-detected Vimentin (Additional file 2).

The percent of E-cadherin, N-cadherin, Mel-cam and EpCAM—positive cells in investigated primary cultures were estimated by flow cytometry using specific antibodies. Data obtained are summarized in Table 2.

**Table 2:** The percent of E-cadherin, N-cadherin, Mel-CAM and EpCAM—positive cells in investigated primary cultures by flow cytometry using specific antibodies

Primary culture	Mel-CAM CD146+	EpCAM CD326+	E-cad CD324+	N-cad CD325+
BrC3e	34.9	98.2	97.0	10.2
BrC4e	33.4	99.0	76.6	ng
BrC5e	23.9	99.0	73.0	ng
BrC6e	42.6	99.0	78.4	ng
BrC4f	96.0	9.7	1.2	85
BrC6f	91.0	20.9	5.6	74
BrCCh3f	93.6	ng	ng	66
BrCCh2e	65.7	60.4	58.1	20.0
BrCCh4e	71.5	97	89	8.8

*E-cad* E-cadherin, *N-cad* N-cadherin, *ng* negative

The expression of indicated markers strongly correlated with cell morphology: fibroblastoid cells demonstrated high Mel-CAM and N-cad with low EpCAM and E-cad whereas epithelial cells demonstrated “reverse” phenotype with low Mel-CAM and N-cad, and high EpCAM and E-cad. Three epithelial cell lines BrC4e, BrC5e and BrC6e expressed no N-cad.

### Sensitivity to chemotherapeutics

**Fig. 5:**
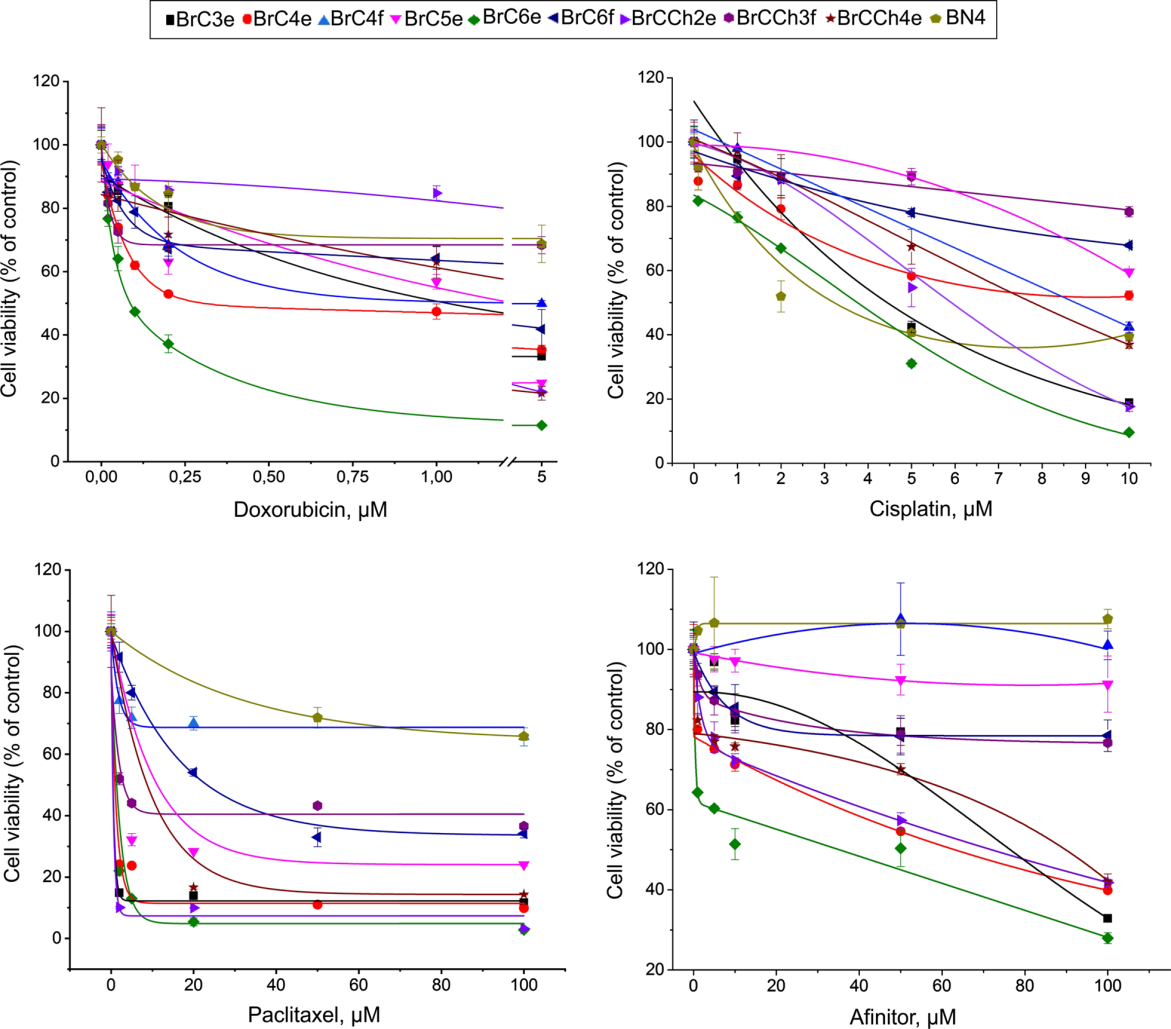
In vitro cytotoxic activity of drugs in personal-derived breast cancer cells. MTT assay was performed 48 h after drugs adding. Cells were seeded at 3000 cells per well and drugs were added [doxorubicin (0.02–5 µM), cisplatin (0.1–10 µM), paclitaxel (2–100 µM), afinitor (1–100 µM)]

Aiming to investigate the sensitivity of established cells to chemotherapeutic, wide-used drugs were selected: paclitaxel and doxorubicin, which are a first-line chemotherapeutics for patients with breast cancer, and cisplatin with afinitor (everolimus) which are out of conventional scheme for breast cancer. Primary cell cultures were exposed to drugs for 48 h and the next MTT-test was made. Concentration-dependent curve of viability revealed sensitive and relatively resistant cell lines (Fig. 5). BrC6e cells were most sensitive to all drugs used. Primary normal BN4 cells were most resistant to doxorubicin, afinitor and paclitaxel but were sensitive to cisplatin. BrCCh3f were resistant to afinitor, doxorubicin and cisplatin. The tendency observed was that fibroblast-like cells were more resistant to drugs used than epithelial-like cells, with some exceptions: BrCCh2e were resistant to doxorubicin and BrC5e were resistant to afinitor and cisplatin. For all drugs used IC50 indexes were calculated (Additional file 3).

### Epithelial-like cancer cells BrC3e, BrCCh2e and BrCCh4e were tumorigenic in SCID mice

Human tumor xenograft models of breast cancer in both MFP (mammary fat pad, orthotopic) and s.c. (subcutaneous, ectopic) environments are two common pre-clinical models. However, both of them show evidence of poor lymphatic drainage that indicates they are far from the ideal cancer model [16]. In order to assess the tumorigenic properties of established cancer cells in vivo, cells were s.c. transplanted into SCID mice. Tumor growth was monitored once a week. Only three cell lines gave rise of tumor growth—BrC3e, BrCCh2e and BrCCh4e (Additional file 4). All tumorigenic cells were with epithelial phenotype and two of them were derived from patients after four courses of chemotherapy. We concluded that EMT markers expression is not advantage for implementation of the tumor-initiating capacity of primary breast cancer cells.

**Fig. 6:**
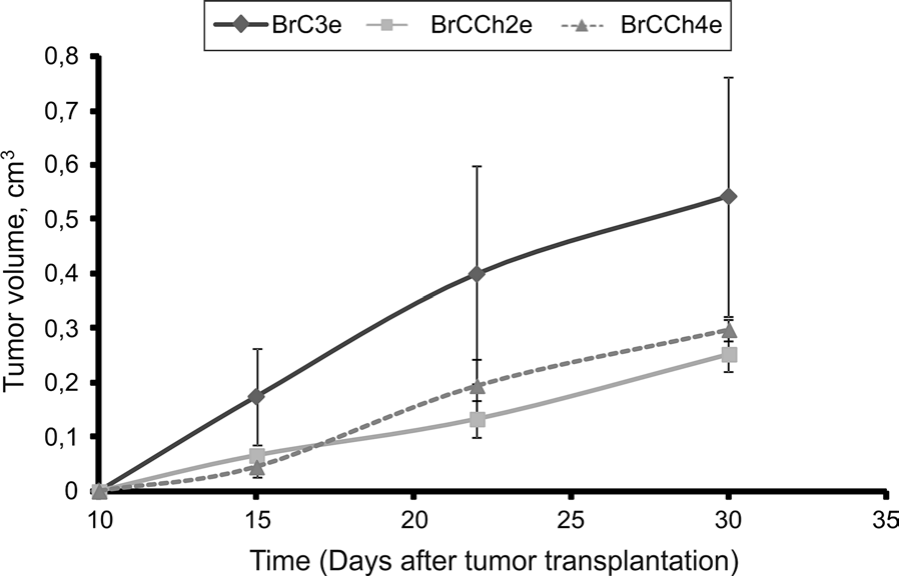
Growth curves of tumors formed by patient-derived breast cancer cells transplanted on SCID mice. Mice were transplanted s.c. with 2 × 10^6^ cells. Tumor volume was monitored once a week during a month

**Fig. 7:**
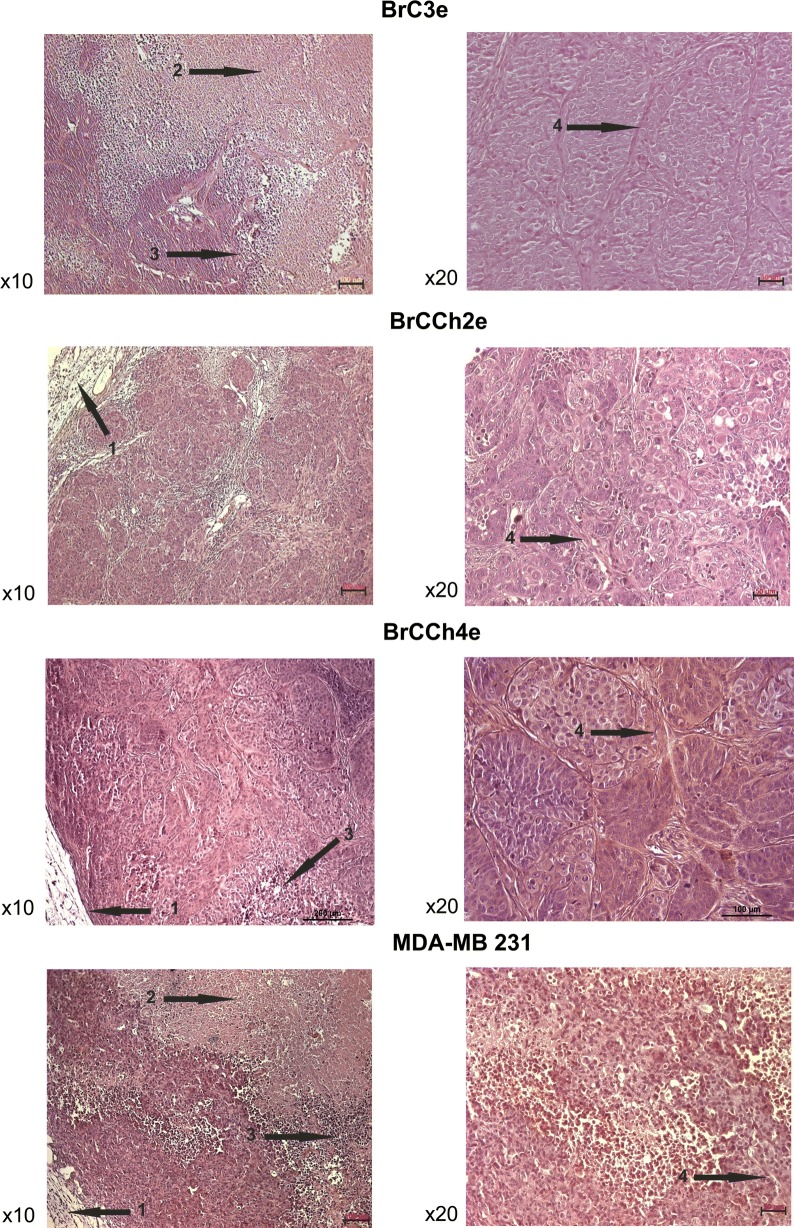
Histological analysis of tumors forming by the patient-derived cancer cells. Tumors were dissected and paraffin blocks were prepared for H&E staining. 1—tissue capsule, 2—necrosis area, 3—inflammation area, 4—fibrosis area

Data obtained demonstrated that the most rapidly growing tumors were initiated by the BrCCh4e cells (Fig. 6). Histologic analysis of tumor tissues revealed the shape of tumors resembling the breast tissue: shapes resembling the lobules of mammary gland were seen (Fig. 7). In contrast to normal breast tissue, developed tumors demonstrated cellular atypism manifested by the changes in nucleus to cytoplasm ratio, hyperchromicity and multiple mitosis. Tissue architecture atypism was evidenced in part by the lack of ducts in the lobules. These alterations are typical for malignant neoplasms when tumor cells proliferate within the duct [17]. Tumor borders are presented by capsule of collagen fibers. Central zonal necrosis was accompanied by lymphocytes infiltration and macrophages accumulation. This pattern was seen in multiple cross-sections. So, tumors formed by BrC3e, BrCCh2e and BrCCh4e kept the visual signs of oncotransformed breast tissue. These tumors were more structured in comparison with tumors forming by human adenocarcinoma MDA-MB-231 cells (Fig. 7).

We have also observed that BrCCh4e cells initiated metastasis when were grafted subcutaneous with colonization of mediastinum lymph nodes (Additional file 5). More interesting that 2 weeks after s.c. transplantation of tumor cells the average size of metastases was comparable with average size of initial tumor node.

### Karyotype analysis of tumorigenic cells

**Fig. 8:**
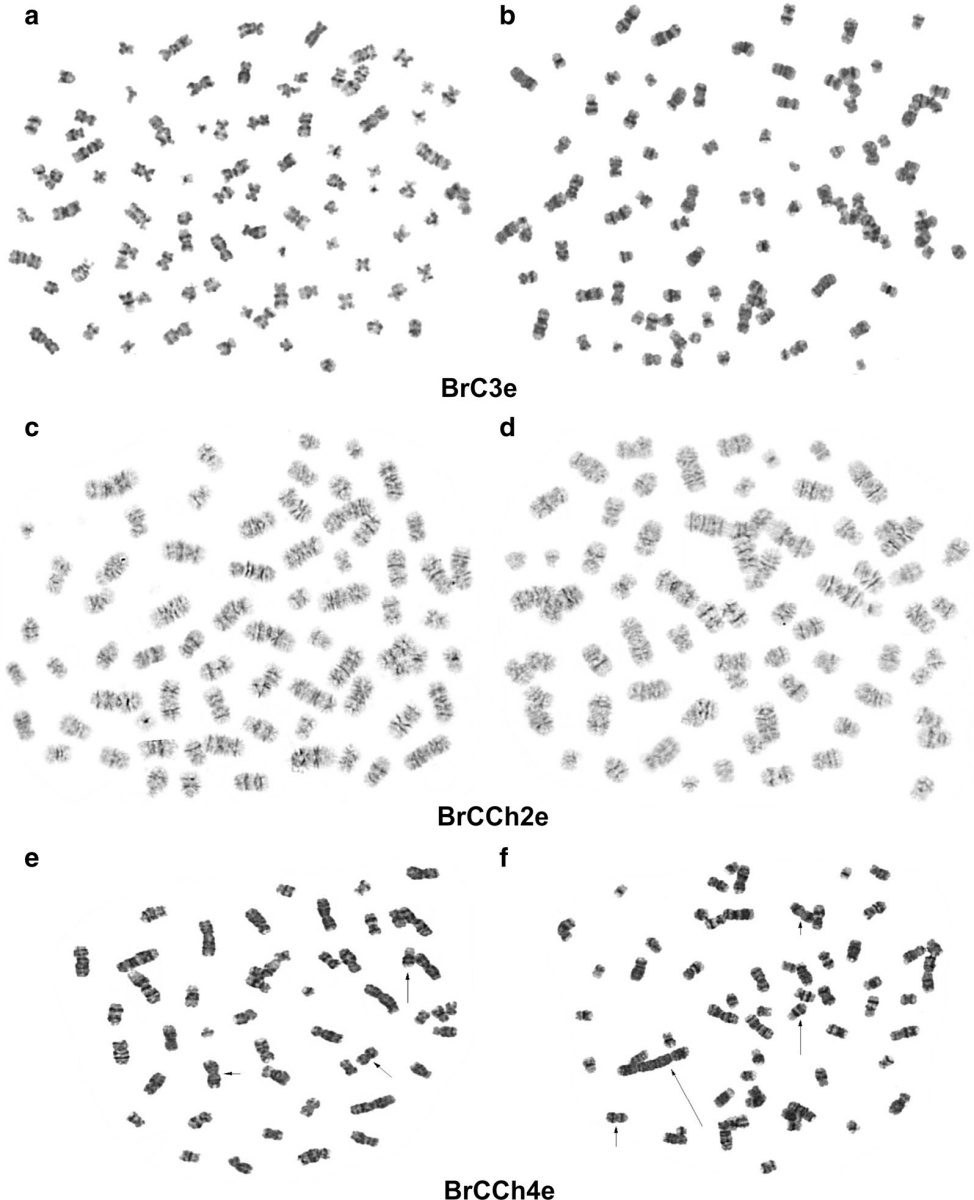
Analysis of GTG-stained chromosomes. Two metaphase plates were in each line. In all metaphase plates, multiple chromosome rearrangements were detected, with some chromosomes in each plate having a pattern of normal GTG-colored human chromosomes

An abnormal chromosome is one of key characteristic of tumor cells. Using colchicine treatment and Giemsa-trypsin banding (GTG-staining), we showed abnormal chromosome sets in all tumorigenic cells. The common changes were multiple chromosome rearrangements but with half of the chromosomes remaining normal with GTG-banding. These cell lines are aneuploid female, with chromosome counts in the near-triploid range. The modal number of chromosomes in BrC3e cells was 79 with ranging from 67 to 91 (Fig. 8). The modal number of chromosomes in BrCCh2e cells was 76 with ranging from 51 to 147. The modal number of chromosomes in BrCCh4e cells was 71 with ranging from 49 to 78 and these cells contain big dicentric chromosome. We observed that the rearrangement of some chromosomes in various metaphases had the same GTG-banding. These findings allow us to assume that once arising in tumor cells such chromosomal rearrangements are inherited through the cell division. However, the observed unique chromosome aberrations indicated that they can be produced under each new round of cell division.

### Ultrastructure of tumorigenic cells

**Fig. 9:**
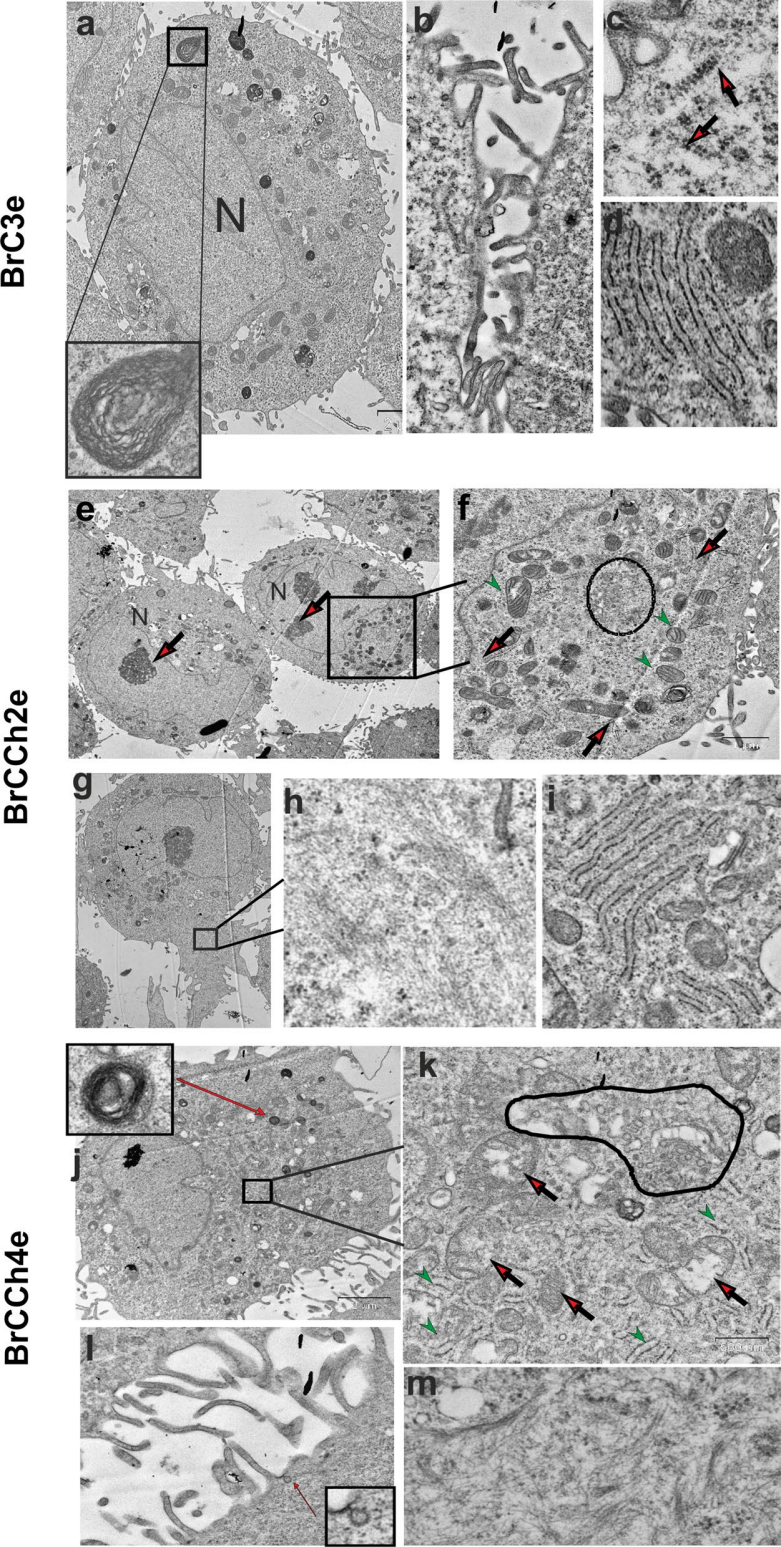
Ultrastructure of established tumorigenic cell lines. Electron microscope images of ultrathin sections. **a** General view BrC3e cell nucleus (N) with myelin-like structures in the sidebar. **b** Fragment of two adjacent cells with numerous protuberances of plasmalemma. **c** Polysomes in the cytoplasm (red arrows). **d** Stack of granular endoplasmic reticulum (ER) **e** General view BrCCh2e cell nucleus (N) with large nucleolus (red arrows), the contour is encircled with cytoplasmic organelles. **f** Cytoplasm fragment with numerous mitochondria (green arrows) and Golgi (circled); ER cisterns are shown by red arrow. **g** Cells with abnormal cytoplasm. **h** Intermediate filaments in the cytoplasm. **i** Stack of granular ER. **j** General view BrCCh4e, myelin-like structure in the sidebar. **k** Cytoplasm fragment with mitochondria (green arrows), stack of granular ER (red arrows) and Golgi (circled), **l** Cells with a broad cytoplasm. **m** Fibers and fiber bundles of intermediate filaments

We next addressed if tumorigenic cells have ultrastructure peculiarities. Ultrastructure analysis revealed low-differentiate phenotype of BrC3e, BrCCh2e and BrCCh4e cells. In cell mass BrC3e and BrCCh4e lines were uniform and were presented by cells with similar morphology while in BrCCh2e the cells with glycogen stores have been seen (Fig. 9). All cells have a roundish big nucleus with no heterochromatin and with presence of 1 or 2 nucleoli. Cell surfaces were rich of coated pits and flask-shaped invagination which are typical signs of microendocytosis. The presence of multivesicular bodies and myelin-like structures in the cytoplasm of investigated cells were detected. Cisterns of granular endoplasmic reticulum (ER) have also been seen in all cell lines.

## Discussion

It is known that during metastasis epithelial cancer cells first undergo EMT producing fibroblast-like cells with migratory and invasive properties [18]. Next step, the conversion of the EMT process to mesenchymal–epithelial transition restores epithelial phenotype of tumor cells in distant metastasis homing sites. In vitro upon successful conditions mesenchymal cells also undergo MET resulting in epithelial carcinoma cells [19]. Nowadays the exact set of stimuli regulating EMT to MET is discussed. We assumed that “pulsed hypoxia” can be one of such stimulus. Usually hypoxia induces EMT with switching in morphology of cancer cells to a fibroblastoid phenotype, and this process is rather fast, taking in vitro about 48 h [20]. In turn, in an oxygen-rich environment, the conversion of EMT to MET was recently demonstrated for pancreatic cancer cells [21]. Take into consideration literature data, it was more impressive in the current work, that the pulsed hypoxic conditions supplemented with the conditioned culture medium have induced transformation of fibroblastoid primary breast cancer cells to epithelial phenotype. This transformation process can be described as a slow and gradual transition, taking up to about 3 weeks. Visible changes of phenotype was accompanied by a switch in expression of essential molecular markers—E-cadherin, N-cadherin, Vimentin, Mel-CAM and EpCAM, which are argued MET-like transformation occurs. While initial fibroblast-like cells were high N-cad-positive and near-negative for E-cad, their daughter epithelial cells were E-cad-positive with no N-cad. Our findings are in good correlation with Kotb et al. who demonstrated that the replacement of E-cadherin by N-cadherin in the mammary glands leads to fibrocystic changes [22]. Further passaging of these epithelial cells under standard conditions did not reverse phenotype to fibroblastoid. These findings wholly support our conclusion that the oxygen concentration is critical for the MET observed. As opposed to migrated metastatic tumor cells in humans, cells in carcinoma metastasis sites lack a mesenchymal phenotype and present epithelial morphology [23]. From this point of view, “pulsed hypoxia” can mimic the conditions that metastatic tumor cells undergo during their travelling in the human organism with the next implantation in a terminal site. Several molecular drivers of MET can be dependent on hypoxia-inducible factors (HIFs) pathways [24]. Besides pulsed hypoxia, future studies need to discover the molecular effectors of shown MET.

**Fig. 10:**
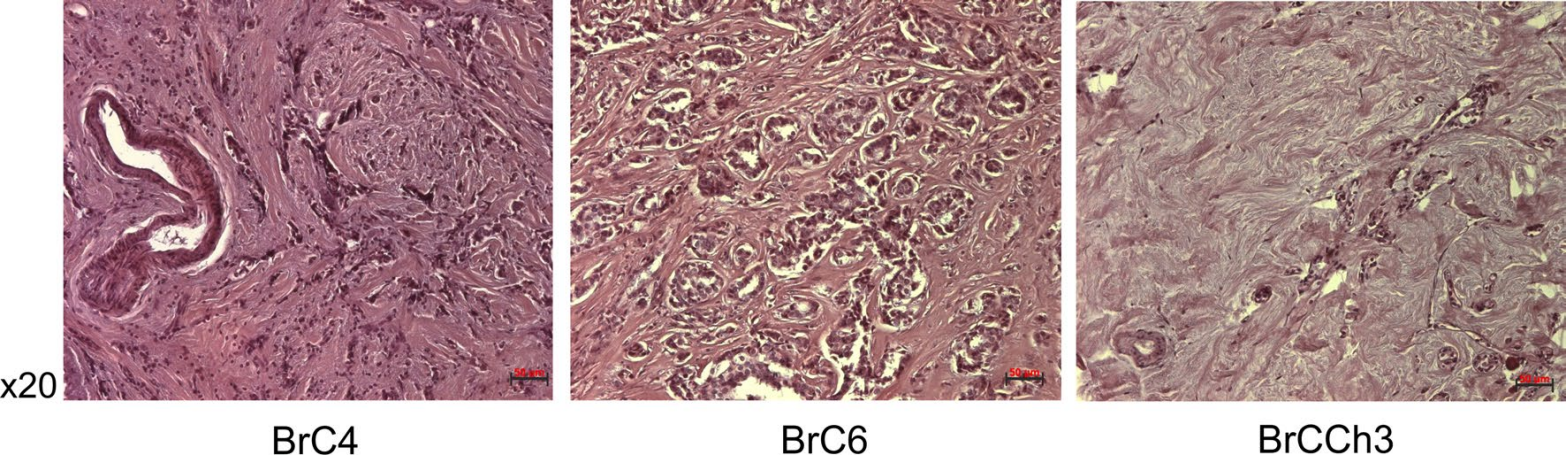
Histological analysis of patient’s tumor samples. All sections are H&E stained. Scale bars represented 50 µm

The lack of conversion of BrCCh3f cells to epithelial phenotype prompted us to re-analyze the original tumor samples. The comparing of histology of BrC4, BrC6, and BrCCh3 tissues (Fig. 10) showed that the BrCCh3 specimen was substantially presented by fibers with single islets of epithelial cells while samples of BrC4 and BrC6 were enriched by cancer-associated fibroblast or breast adipocytes. Therefore, we concluded that the source of cancer tissue is crucial for the establishing of the breast cancer personal cell lines by the collagenase I method.

Resistance to chemotherapeutic drugs is one of key reasons that leads to inefficient treatment of breast cancers. Cell models with chemoresistance are a powerful tool for investigation of molecular mechanism of such phenomenon. Here, three of nine cell lines were obtained from doxorubicin-treated patients that can stimulate chemoresistance. Indeed, two cell lines—BrCCh2e and BrCCh3f were relatively resistant to doxorubicin and they can be helpful models for the further research of genetic or other molecular basis for the resistance. We observed that these cell lines expressed low Ki-67 which means a low proliferation rate and it can partly explain weak sensitivity to doxorubicin.

The analysis of tumor-initiating capacity of established cancer cell lines showed that neither fibroblast-like cells nor epithelial-like cells originated from these cells produced tumors in mice. Nowadays, the question of what cancer cells—with EMT or with MET phenotype—promote tumor formation in vivo is still discussed. There is evidence for both conceptions. Several studies have shown that transformed human mammary epithelial cells, that have undergone an EMT, form tumors more efficiently than none-EMT cells [25]. However, there is evidence that EMT and tumor-initiating capacity may not be directly linked in breast cancer cell lines [7]. These findings wholly support our conclusion that breast cancer cells, that had undergone EMT, don’t have a tumor-initiating capacity. On the other hand, breast cancer cells that had undergone MET form palpable tumors with delayed time and displayed slower tumor growth in vivo suggesting that the MET process does not lead to inhibition or loss of tumor-initiating capacity of breast cancer cells [7]. Our failure in growing tumors from the MET-undergo BrC4e and BrC6e cells in vivo, in part can be explained by the fact that the observation period of tumor growth was too short for such cells. Finally, we agree with *Xie*, who declares that in some breast cancer cell lines only the inherent ability of cancerous cells is responsible for tumor-initiating capacity, not EMT or MET status [7].

It is known that breast cancer cells over-expressing ErbB2 depend on its activity for proliferation. BrC4f and BrC6f showed the highest HER2 and HER3 receptor tyrosine kinases co-expression and then they became epithelial, BrC4e and BrC6e completely lost their HER3. In turn, all established epithelial cells expressed the HER2 receptor. Among tumorigenic cells, BrC3e and BrCCh2e were HER3-negative and demonstrated the similar HER2 expression [13]. BrCCh4e cells expressed the highest HER3 among established epithelial cells and it can promote the metastasis observed in BrCCh4e tumor-bearing mice. It is known that the HER2 and HER3 receptor tyrosine kinases co-expression can enhance invasiveness and motility of breast cells [26]. Because of HER2/HER3 dimer functions as an oncogenic unit, the loss of HER3 in HER2-dependent breast cancer cells reduces cell proliferation [27]. Comparing other tested markers in established epithelial cells to reveal the differences between tumorigenic and non-tumorigenic epithelial cells, we can conclude that non-tumorigenic cells did not express N-cad.

Among the established tumorigenic cell lines, all of them demonstrated aneuploid chromosome numbers with multiple complex rearrangements that makes karyotypes unstable. More interesting, we detected big dicentric chromosome in BrCCh4 cells. The proposed origin of such chromosome rearrangements is that they errors in the processes of replication, recombination and cytokinesis. Dicentric chromosomes can be seen quite often in cancer cells and even a single dicentric chromosome can drive further genome rearrangement and neoplastic conversion [28]. Moreover, we have shown that only BrCCh4e cells produced metastases. These cells expressed high Ki-67, the proliferation-related protein (Fig. 4b), that is typical for poorly differentiated tumors. It is known that all metastatic tumors expressed at least an intermediate Ki-67, that is in good agreement with our data [29].

In breast cancer patients metastasis usually occurs in regional lymph nodes, bone, lung, liver and brain [30]. Lymph nodes metastasis in association with tumor size is a powerful indicator of poor prognosis in mammary carcinomas [31]. To mimic breast cancer metastasis, the orthotopic or ectotopic implantation of cancer cells as well as direct cancer cells injections into tail vein or left ventricle of the heart are usually used [32, 33]. Overviewing well-characterize and wide-used human breast cancer cell lines, only MCF7 and T47D cells generated lymph node- and lymph vessel metastasis in Nude or SCID mice [3]. MCF7 and T47D cells produce metastasis only 12 weeks after the orthotropic transplantation on immunodeficient mice, and in addition MCF7 cells require oestrogene supplementation for tumor growth [31]. NOD *scid* gamma mice were shown as a new mouse model for human breast cancer metastasis where primary MDA-MB-231, MDA-MB-436 and MCF7-formed tumors produced metastases in axillary, inguinal or mesenteric, but not mediastinum lymph nodes [34]. Established BrCCh4e cells, growing well without external supplementation with potency to induce rapid metastasis in mediastinum lymph nodes can be a helpful model to study the molecular mechanisms underlying breast cancer metastasis to lymph nodes and for downstream applications.

## Conclusions

The developed BC cells represent a tool for translational research. Our findings demonstrate that “pulsed hypoxia” induces transformation of primary fibroblast-shaped breast cancer cells to epithelial-like cells and both of these cultures—induced and original—don’t show tumor-initiating capacity. It seems that “pulsed hypoxia” can mimic the conditions that metastatic tumor cells undergo during their travelling in the human organism with the next implantation in a terminal site. Our study finds a links between molecular characteristics of patient-derived breast cancer cells and their tumorigenic potential. We suppose that in some breast cancer cell lines only the inherent ability of cancerous cells is responsible for tumor-initiating capacity, not EMT or MET status. The developed BC cells metastasizing to mediastinum lymph nodes are a relevant model for downstream applications.

## Additional files

**Additional file 1.** mRNA expression level of breast tumor related genes in established cell lines.

**Additional file 2.** Western blot analysis of Vimentin in patient-derived cancer cells.

**Additional file 3.** The IC50 for doxorubicin, cisplatin, paclitaxel and afinitor in patient-derived cancer cells.

**Additional file 4.** Tumorigenicity of patient-derived breast cancer cells in immunodeficient mice. Representative images of tumor-bearing mice.

**Additional file 5.** Representative image of metastasis in mediastinum lymph node of BrCCh4e tumor-bearing mouse.

## Abbreviations

ASCO: American Society of Clinical Oncology
BC: breast cancer
Cyp19: aromatase cytochrome P450
E-cad: E-cadherine
EMT: epithelial–mesenchymal transition
EpCAM: epithelial cell adhesion molecule
ErbB2: gene receptor tyrosine-protein kinase
ER: endoplasmic reticulum
ERs: estrogen receptors
HER: human epidermal growth factor receptor
Mel-CAM: melanoma cell adhesion molecule
MET: mesenchymal-to-epithelial transition
MTT: 3-(4,5-dimethylthiazol-2-yl)-2,5-diphenyltetrazolium bromide
GTG-staining: Giemsa-trypsin banding
N-cad: N-cadherine
PR or PGR: progesterone receptor
Ki-67: nuclear protein being associated with cellular proliferation
