# Supplementary figures and images for: Establishment of primary human breast cancer cell lines using “pulsed hypoxia” method and development of metastatic tumor model in immunodeficient mice
Source: Cancer Cell Int. 2019 Feb 28;19:46. doi: 10.1186/s12935-019-0766-5 (PMC6394017; doi:10.1186/s12935-019-0766-5)

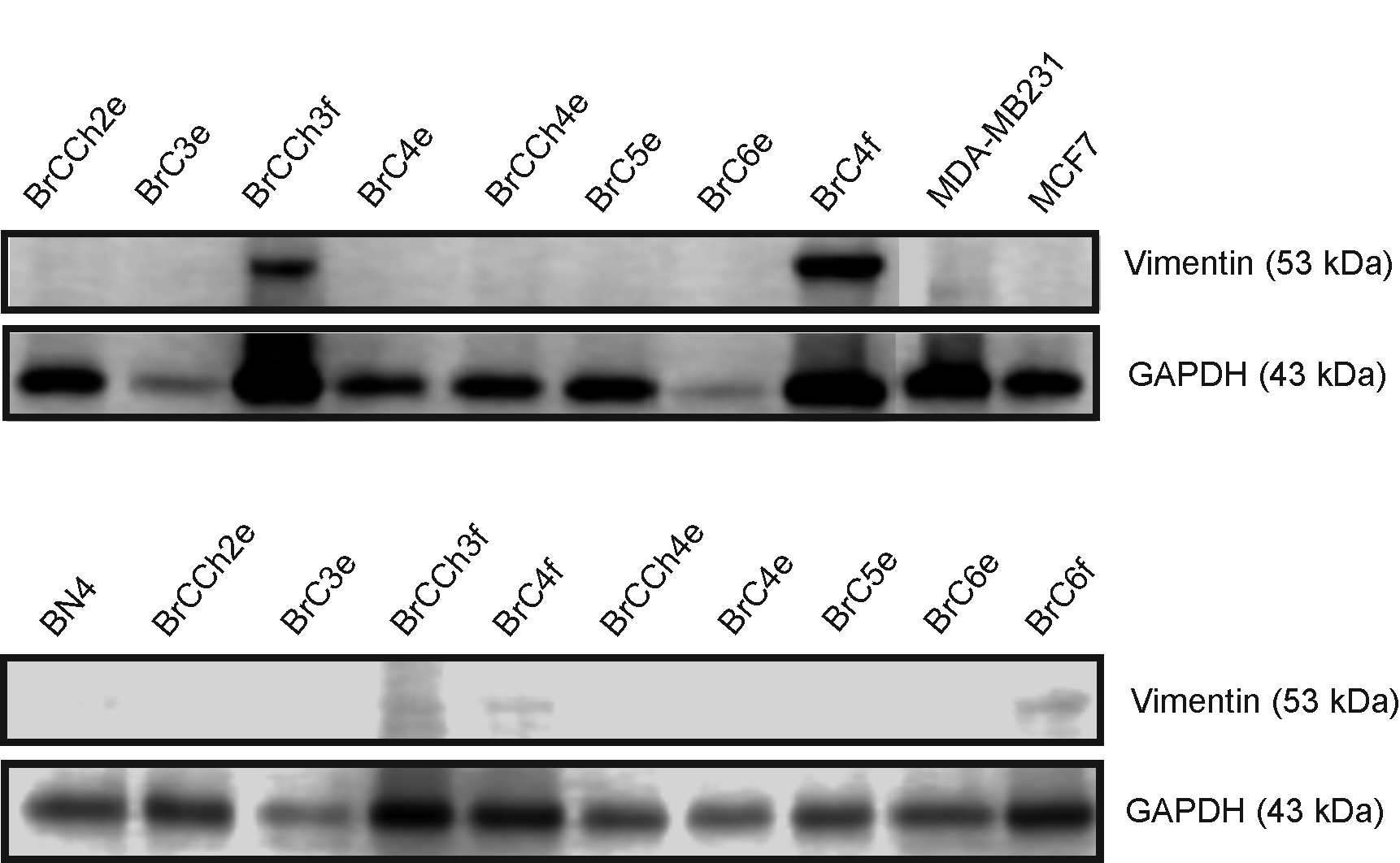

Supplement: Supplementary file 2 — Additional file 2. Western blot analysis of Vimentin in patient-derived cancer cells. [file 12935_2019_766_MOESM2_ESM.tif]

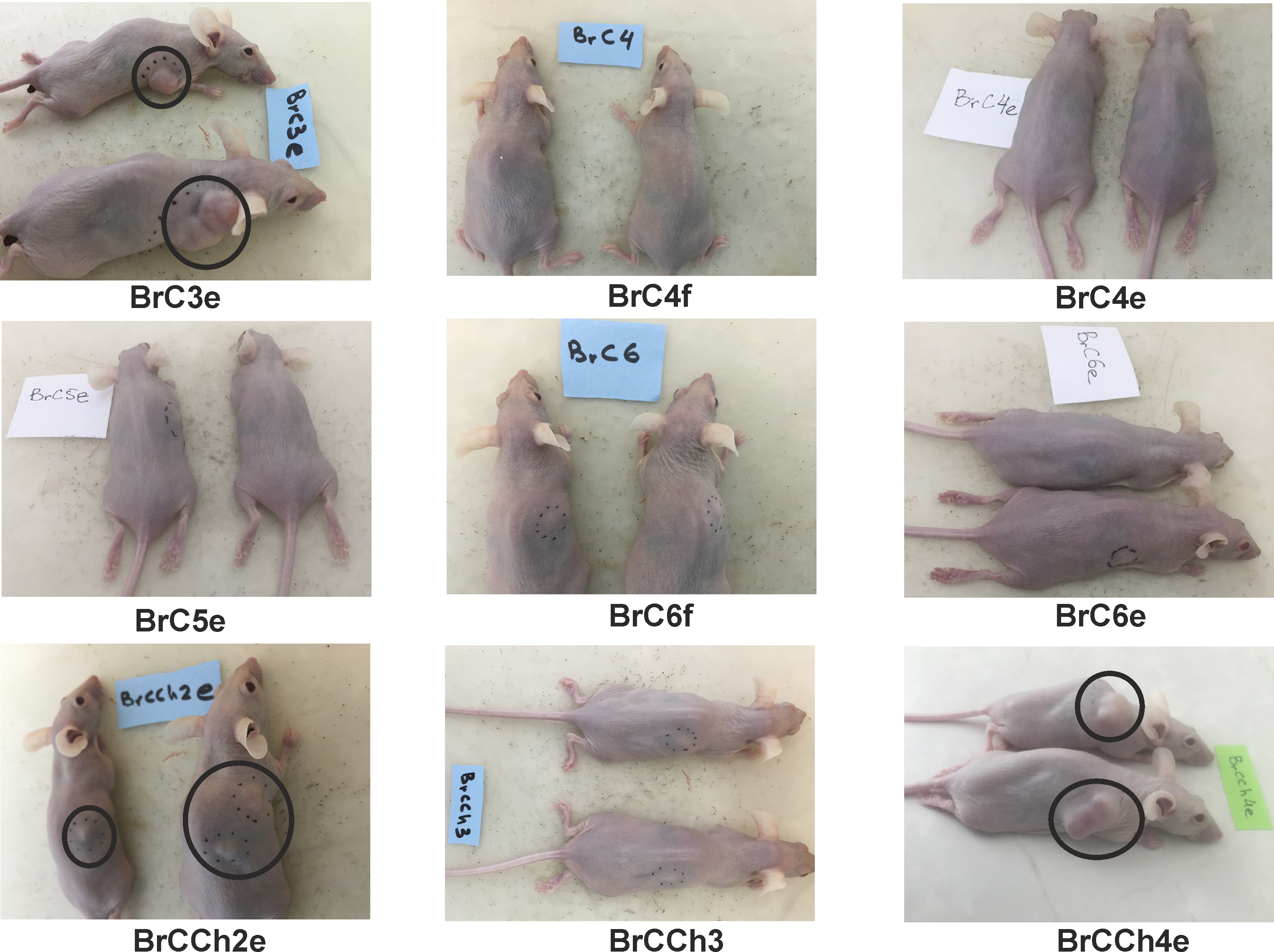

Supplement: Supplementary file 4 — Additional file 4. Tumorigenicity of patient-derived breast cancer cells in immunodeficient mice. Representative images of tumor-bearing mice. [file 12935_2019_766_MOESM4_ESM.tif]

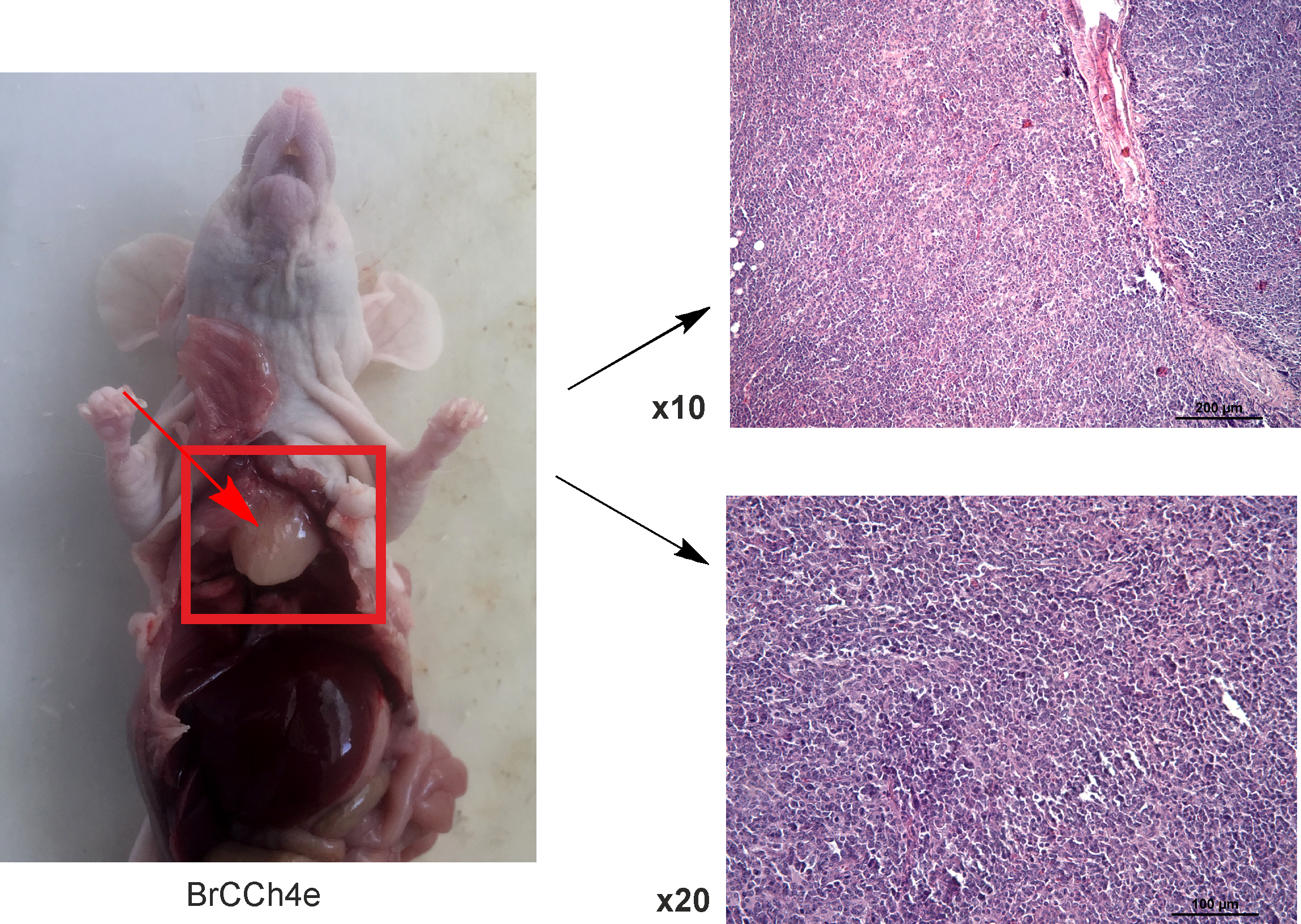

Supplement: Supplementary file 5 — Additional file 5. Representative image of metastasis in mediastinum lymph node of BrCCh4e tumor-bearing mouse. [file 12935_2019_766_MOESM5_ESM.tif]
