# Supplementary material for: Establishment of primary human breast cancer cell lines using “pulsed hypoxia” method and development of metastatic tumor model in immunodeficient mice
Source: Cancer Cell Int. 2019 Feb 28;19:46. doi: 10.1186/s12935-019-0766-5 (PMC6394017; doi:10.1186/s12935-019-0766-5)
Supplement: Supplementary file 3 — Additional file 3. The IC50 for doxorubicin, cisplatin, paclitaxel and afinitor in patient-derived cancer cells. [file 12935_2019_766_MOESM3_ESM.docx]

Additional 3

The half-maximal inhibition (IC50) for doxorubicin, cisplatin, paclitaxel and afinitor

|  | IC50 values of drug (µМ) | | | |
| --- | --- | --- | --- | --- |
| Primary culture | Doxorubicin | Cisplatin | Paclitaxel | Afinitor |
| BrC3e | 5.6 | 4.7 | <2 | 64.9 |
| BrC4f | 5.7 | 9.7 | not reached | not reached |
| BrC4e | 0.7 | not reached | <2 | 62.5 |
| BrC5e | 0.9 | not reached | <2 | not reached |
| BrC6f | 0.4 | not reached | 29.6 | not reached |
| BrC6e | 0.1 | 1.7 | <2 | 13.2 |
| BrCCh2e | 3.0 | 6.5 | <2 | 67.9 |
| BrCCh3f | not reached | not reached | 9.3 | not reached |
| BrCCh4e | 0.9 | 7.3 | <2 | 164.3 |
| BN4 | not reached | 3.4 | not reached | not reached |
